# Exploring genetic variants in premature ovarian insufficiency: a cohort-based investigation of monogenic etiologies

**DOI:** 10.1210/jendso/bvag215

**Published:** 2026-10-28

**Authors:** Asma Sassi, Xavier Peyrassol, Martina Marangoni, Alexander Gheldof, Sonia Van Dooren, Beatrice Alvaro Mercadal, Axelle Pintiaux, Caroline Antoine, Marc Abramowicz, Julie Désir, Anne Delbaere

**Affiliations:** Department of Obstetrics and Gynecology, Fertility Clinic, Hôpital Universitaire de Bruxelles (H.U.B), CUB Hôpital Erasme, Université Libre de Bruxelles (ULB), Brussels 1070, Belgium; Department of Human Genetics, Hôpital Universitaire de Bruxelles (H.U.B), CUB Hôpital Erasme, Université Libre de Bruxelles (ULB), Brussels 1070, Belgium; Department of Human Genetics, Hôpital Universitaire de Bruxelles (H.U.B), CUB Hôpital Erasme, Université Libre de Bruxelles (ULB), Brussels 1070, Belgium; Clinical Sciences-Research Group Reproduction and Genetics, Centre for Medical Genetics, Medical Genetics, Vrije Universiteit Brussel VUB, Universitair Ziekenhuis Brussel UZ Brussel, Brussels 1090, Belgium; Clinical Sciences-Research Group Reproduction and Genetics, Centre for Medical Genetics, Medical Genetics, Vrije Universiteit Brussel VUB, Universitair Ziekenhuis Brussel UZ Brussel, Brussels 1090, Belgium; Clinical Sciences-Research Group Reproduction and Genetics, Brussels Interuniversity Genomics High Throughput Core (BRIGHTcore), Vrije Universiteit Brussel (VUB), Universitair Ziekenhuis Brussel UZ Brussel, Brussels 1090, Belgium; Centre for Medical Genetics, Medical Genetics, Clinical Sciences-Research Group Reproduction and Genetics, Vrije Universiteit Brussel VUB, Universitair Ziekenhuis Brussel UZ Brussel, Brussels 1090, Belgium; Gynecology, Fundació Puigvert de Barcelona, Hospital de Sant Pau UAB, Barcelona 08025, Spain; Department of Gynecology, Centre Hospitalier Universitaire de Liège, Liège 4000, Belgium; Department of Gynecology, Hôpital Universitaire Saint-Pierre, Brussels 1000, Belgium; Human Genetics, Institute of Interdisciplinary Research in Human and Molecular Biology (IRIBHM), Université Libre de Bruxelles (ULB), Brussels 1070, Belgium; Department of Human Genetics, Hôpital Universitaire de Bruxelles (H.U.B), CUB Hôpital Erasme, Université Libre de Bruxelles (ULB), Brussels 1070, Belgium; Department of Obstetrics and Gynecology, Fertility Clinic, Hôpital Universitaire de Bruxelles (H.U.B), CUB Hôpital Erasme, Université Libre de Bruxelles (ULB), Brussels 1070, Belgium

**Keywords:** premature ovarian insufficiency, hypergonadotropic hypogonadism, genetics, next-generation sequencing, gene disease relationship scoring

## Abstract

**Context:**

Premature ovarian insufficiency (POI), affecting about 3.5% of women under 40, is marked by menstrual irregularities, amenorrhea, and heterogeneous pubertal development. Although next-generation sequencing (NGS) has expanded the list of POI-associated genes, most cases remain unexplained, and the clinical diagnostic value of gene panels is still insufficiently assessed.

**Objective:**

This study combines a cohort-based analysis with a critical methodological reassessment of NGS diagnostic yield in POI.

**Design:**

Prospective multicenter observational cohort study (2016-2023).

**Setting:**

Three Belgian academic hospitals.

**Patients:**

One hundred sixty unrelated women with idiopathic POI and 716 female controls.

**Intervention:**

Targeted NGS of 155 POI-associated genes. Variants were classified following American College of Medical Genetics and Genomics/Association for Molecular Pathology guidelines, and genes were evaluated using the latest gene–disease relationship (GDR) scoring framework.

**Main outcome measures:**

Identification of pathogenic/likely pathogenic (P/LP) variants (primary outcome). Diagnostic yield of the gene panel based on genes with strong evidence (secondary outcome).

**Results:**

Pathogenic/likely pathogenic variants were identified in 19.3% of patients; however, only 7.5% were considered clinically relevant based on gene validity and inheritance patterns. Limiting analysis to the 40 genes with the strongest GDR evidence yielded a 4.4% diagnostic rate. Adding 10 genes supported by recent literature increased the yield to 7.5%. In patients with primary amenorrhea, the diagnostic rate reached 15.4%. These findings highlight the gap between variant burden and true diagnostic yield in POI.

**Conclusion:**

Targeted NGS panels provide valuable clinical insight into POI genetics but should be complemented with broader genomic approaches for comprehensive diagnosis.

Premature ovarian insufficiency (POI) is a heterogeneous condition affecting about 3.5% of women under 40 (1). It is defined by primary amenorrhea (PA) or secondary amenorrhea (SA) or persistent oligo-amenorrhea for ≥4 months, associated with hypergonadotropic hypogonadism (2).

According to recent international guidelines, a single follicle-stimulating hormone (FSH) value >25 IU/L is sufficient for diagnosis when clinical features are suggestive, although a second measurement may be useful in uncertain situations (1). Prevalence varies across populations and is influenced by ethnic and socioeconomic factors (2-4). Etiologies include autoimmune, infectious, toxic, and genetic causes (5-8), yet a substantial proportion of cases remain idiopathic (8-10). Genetic factors are estimated to contribute to at least 20% of POI cases (5, 11).

## Guidelines for genetic assessment

The 2024 international guideline jointly developed by European Society of Human Reproduction and Embryology (ESHRE), American Society for Reproductive Medicine, International Menopause Society, and Centre for Research Excellence in Women's Health in Reproductive Life recommends offering genetic testing to all women with noniatrogenic POI (1). Genetic evaluation should be initiated only after the clinical diagnosis is confirmed. First-line testing includes karyotype analysis and screening for the FMR1 premutation (OMIM: 309550). If these investigations are normal, second-line assessment involves targeted next-generation sequencing (NGS) panels, with variant interpretation performed according to the American College of Medical Genetics and Genomics/Association for Molecular Pathology (ACMG/AMP) standards. All genetic testing must be preceded by appropriate genetic counseling and undertaken with documented informed consent (1).

## Genetic architecture of POI

The genetic background of POI includes chromosomal abnormalities, FMR1 premutations, and rare variants identified in both syndromic and nonsyndromic forms (8, 9, 12-14). With the advent of NGS, the number of genes associated with POI has expanded considerably (11, 13-22). These genes participate in essential biological pathways, including meiosis and DNA repair, ovarian and follicular development, metabolism, steroidogenesis, mitochondrial function, apoptosis, autophagy, and immune regulation (9, 12, 21, 23). Next-generation sequencing studies have also suggested oligogenic or polygenic mechanisms (24, 25), and several pleiotropic variants have been reported in women presenting with apparently isolated POI (21, 26, 27). Although more than 200 genes have been implicated, the strength of evidence is highly variable, with many genes supported by limited or inconsistent data (13). To address this heterogeneity, the gene–disease relationship (GDR) scoring system provides an objective framework to assess the clinical validity of POI-associated genes (13, 28).

## Clinical utility of genetic testing

Genetic testing refines the diagnosis of POI by identifying underlying etiologies, informing prognosis, guiding fertility planning and individualized management, and supporting family screening, thereby complementing clinical assessment (1, 11, 13-22). In clinical practice, targeted sequencing of known POI-associated genes either through custom-designed gene panels or in silico panels generated from whole-exome sequencing (WES) can be used as a diagnostic approach (11, 18, 21, 29-31).

While genetic testing is clearly valuable for relatives, it also provides important and actionable information for the patient herself, with direct implications for reproductive counseling, clinical evaluation, and long-term management. First, a molecular diagnosis can be relevant for reproductive counseling and clinical decision-making at the individual level. For instance, monogenic forms, such as some variants in the *FSHR* gene, may have specific therapeutic implications, including potential eligibility for tailored approaches such as in vitro oocyte maturation (32). In addition, genetic findings may reveal underlying pleiotropic or syndromic conditions or be associated with long-term risks, such as malignancy, thereby supporting individualized clinical evaluation and follow-up (21, 27, 28).

## Ethical considerations and need for expanded evidence

Many genetic variants remain of uncertain significance (VUS), and the age at POI onset can vary widely, even among individuals carrying the same variant. This complexity is amplified by the heterogeneity of underlying etiologies and by the well-documented variability in penetrance and expressivity across multiple POI-associated genes, making clinical interpretation particularly challenging. These uncertainties underscore the need for clear communication, long-term follow-up, and appropriate counseling (1). In this context, informed consent and structured pre- and posttest genetic counseling are essential to help patients understand the potential implications of genetic testing, including uncertain or incidental findings, psychological impact, and familial consequences. Despite significant advances, many genetic causes of POI remain unidentified, highlighting the need for comprehensive, evidence-based gene panels to improve diagnostic yield and uncover novel pathogenic mechanisms (11, 13-22, 29-31).

## Methodological limitations in published studies

Interpreting genetic variants in POI remains particularly challenging due to multiple methodological limitations across published studies. Reported diagnostic yields may be overestimated when ACMG/AMP criteria are not consistently applied or when VUS and candidate genes with limited evidence are included. Additional biases stem from the duplication of familial cases, cohort differences in ethnicity and clinical characteristics, and the substantial variability in both the number and selection of genes sequenced across studies. Moreover, many cohorts include heterogeneous phenotypes, combining syndromic and nonsyndromic POI and variable proportions of severe presentations such as PA, making cross-study comparisons difficult. This phenotypic heterogeneity underscores the need for harmonized definitions and more homogeneous phenotype stratification in future genetic investigations of POI (11, 18, 21, 29-31).

## Study context and objectives

To address methodological limitations and reduce the impact of phenotypic and technical heterogeneity in previous studies, we developed an in silico POI gene panel of 155 genes previously associated with the disorder in humans. This panel was applied to a cohort of 160 unrelated women with apparently nonsyndromic POI, predominantly of Caucasian origin, with additional participants of North African and South African origin. The primary objective was to identify pathogenic/likely pathogenic (P/LP) variants across this curated gene panel. The secondary objective was to assess the diagnostic yield of this approach in clinical practice by focusing on the most relevant POI genes according to the GDR scoring system proposed by Van der Kelen et al (13) and adopted by Female Reproductive Genetics Initiative (FeRGI)/ESHRE, supplemented with updated evidence from the literature.

## Materials and methods

### Study design and setting

This prospective cohort study was conducted between 2016 and 2023 across 3 Belgian academic and university hospitals: Hôpital Universitaire de Bruxelles-Hôpital Erasme, Hôpital Universitaire Saint-Pierre, and Hôpital de la Citadelle, as well as at the Institut de Pathologie et de Génétique in Gosselies, Belgium. The study was approved by the ethics committee of Hôpital Erasme (study registration number: P2016/196/CCB B406201628264).

### Participants

Eligible participants were women diagnosed with idiopathic POI, according to the 2016 ESHRE guidelines (2): oligomenorrhea or amenorrhea for at least 4 months before age 40, with elevated FSH values on 2 occasions more than 4 weeks apart. Women with autoimmune POI (positive antiadrenal antibodies), iatrogenic causes (ovarian surgery, chemotherapy, radiotherapy), abnormal karyotypes, or FMR1 premutations were excluded. All patients provided written informed consent. The control cohort included 716 women from the Highlander NGS control database at Erasme Hospital, all of whom also provided written informed consent for the use of their sequencing data in medical research.

### Gene panel construction and gene–disease evidence evaluation

Premature ovarian insufficiency–associated genes were identified through a PubMed search up to January 2023 using the keywords POI, gonadal dysgenesis, hypergonadotropic hypogonadism, ovarian failure, genetics, and NGS. An in silico panel of 155 genes previously linked to POI was established (Tables S1 and S2) (33). Genes were classified as either diagnostic genes (high relevance) or candidate genes (lower relevance). Gene relevance was assessed using the GDR scoring system by Van der Kelen et al (13), which integrates the number of unrelated affected individuals, the number of P/LP variants, independent publications confirming earlier findings, and available biological validation. Based on the GDR scoring system, genes were categorized into 5 evidence levels for POI causality: no evidence (<3), limited (3 to <9), moderate (9 to <13), strong (13 to <16), and definitive (16-17) (13, 28). Genes with a GDR score ≥9 were considered the most relevant and classified as diagnostic POI genes (40 genes). Because the GDR scoring system incorporated evidence only up to November 2021, we conducted an updated review of genes with GDR <9 or not previously classified. A new PubMed search was performed for articles published between December 2021 and February 2025 using the following MeSH terms: (“next generation sequencing” OR “Gene Panel” OR “Gene Variant” OR “Whole Exome Sequencing”) AND (“Primary Ovarian Insufficiency” OR “Premature Ovarian Insufficiency” OR “Premature Ovarian Failure” OR “Ovarian Dysgenesis” OR “Gonadal Dysgenesis” OR “Early Menopause”). Each gene was then reevaluated individually using its gene name combined with the same MeSH terms. We considered a gene with GDR <9 to be clinically relevant if at least 2 newly reported unrelated patients carried P/LP variants consistent with the known mode of inheritance (Table S3) (33). Genes mentioned in the literature but not included in Van der Kelen et al were documented but not classified as diagnostic genes, since they had not undergone formal GDR assessment and were therefore retained as candidate genes.

### Updated evidence supporting additional diagnostic POI genes

Following a comprehensive literature review covering publications from December 2021 to February 2025, 31 studies were identified reporting VUS, LP, and P variants across 50 genes among the remaining 115 genes evaluated. In total, 122 P/LP variants were reported in 125 unrelated POI patients. Based on the inheritance patterns described for these genes, evidence supports a potential causal role for 10 genes that previously had GDR scores <9 (Table S3) (33). These newly supported genes include *AMH* (2 unrelated patients) (34); *ATM* (7 patients) (21, 22, 34); *BRCA2* (3 patients) (21); *CPEB1* (2 patients) (22); *HAX1* (2 patients) (35); *MEIOB* (2 patients) (36); *NBN* (2 patients) (21, 22); *POLG* (9 patients) (22, 31, 34); *PRDM1* (3 patients) (22); and *TP63* (13 patients) (21, 22, 37-40) (Table S3) (33). Based on this updated evidence, these 10 genes were incorporated into the diagnostic gene set. Consequently, the diagnostic panel now comprises 50 genes (40 genes with GDR ≥9 and 10 newly supported genes). The remaining 105 genes were retained as candidate genes. Among these, heterozygous P/LP variants were identified in *TSC2* (*n* = 5), WDR62 (*n* = 4), *BLM* (*n* = 7), and *RECQL4* (*n* = 13). Although *BLM* and *RECQL4* have been implicated in autosomal recessive (AR) syndromic POI (9), these 4 genes were not further considered in the analysis because their GDR scores have not yet been formally evaluated (Table S3) (33).

### High-throughput sequencing

Genes included in the in silico panel were filtered from Mendeliome sequencing (2016-2022) and WES (June 2022 to June 2023). Mendeliome was initially used in our genetics department (41) before being replaced by WES in 2022. The Mendeliome panel includes 4867 OMIM-listed genes associated with monogenic disorders and was sequenced at the Brussels Interuniversity Genomics High Throughput Core (www.brightcore.be). For Mendeliome sequencing, genomic DNA (1 µg) was fragmented (∼220 bp) using a Covaris M220 ultrasonicator, libraries were prepared with the KAPA HyperPrep Kit (Roche), enriched with Roche NimbleGen SeqCap EZ Choice XL probes, and sequenced in paired-end mode (2 × 100 or 2 × 125 bp) on Illumina HiSeq 1500 or NovaSeq 6000 platforms. Reads were aligned to hg19 using BWA-MEM, processed with SAMtools and Picard, optimized with Genome Analysis Toolkit (GATK), and coverage was assessed using SAMtools and an in-house R script. Variant calling (GATK) and annotation were performed using the Highlander pipeline (https://sites.uclouvain.be/highlander). Whole-exome sequencing was performed by the Department of Human Genetics, Hôpital Universitaire de Bruxelles (H.U.B.), CUB Hôpital Erasme, Brussels, Belgium using the Twist Comprehensive Exome Panel (Twist Bioscience), with sequencing (2 × 150 bp paired-end) on the NovaSeq 6000 platform at KU Leuven. Reads were aligned to hg38, and variant annotation and analysis were also performed using the Highlander pipeline.

### Variant annotation, filtering, and prioritization

Variant annotation and filtering were performed using the Highlander pipeline (https://sites.uclouvain.be/highlander). Within Highlander, SnpEff, an open-source tool for functional variant annotation, is natively integrated and automatically generates snpeff_effect and snpeff_impact annotations, enabling prediction of functional consequences and impact levels (42). Filtering included sample identifiers and restriction to the predefined list of 155 POI-associated genes. Quality filters consisted of PASS status (variant meeting the predefined quality criteria) according to standard GATK criteria (43), a minimum read depth ≥10, and exclusion of variants flagged as potential sequencing artifacts by Highlander's internal QC module. Variants were further prioritized based on allele frequency, using the maximum minor allele frequency (MAF) across population databases integrated into Highlander, as well as our internal NGS control database (>6125 individuals). Only variants with MAF ≤1% were retained, following the conventional distinction between common single-nucleotide polymorphisms (≥1%) and rare variants (<1%) (44, 45). The first 150 patients and all controls were processed using Highlander version 16.1, incorporating Mendeliome sequencing data aligned to hg19. The same variant-filtering criteria were consistently applied. The remaining 10 patients were analyzed using Highlander version 17.18, incorporating WES data aligned to hg38. Full details on filtering parameters are provided in the supplemental material (“Highlander filters”) (46). All 155 POI-associated genes were analyzed in both Mendeliome and WES datasets. For each gene, detailed coverage metrics including transcript identifiers; exon-level mean depth; and the percentage of targeted bases covered at ≥30× for Mendeliome and ≥30× and ≥100× for WES are provided in Table S2 (33). Mean coverage was 229× for Mendeliome and 272× for WES, with >95% of targeted bases covered at ≥30× on both platforms.

### Variant classification

Variants were classified according to the ACMG/AMP 2015 guidelines (47) and ClinVar (https://www.ncbi.nlm.nih.gov/clinvar/docs/clinsig/). Truncating variants fulfill PVS1 criteria, whereas nontruncating variants required integration of additional supporting evidence. Variant interpretation was refined using several external resources, including InterVar (https://wintervar.wglab.org/), VarSome Clinical (https://varsome.com/), Franklin by Genoox (https://franklin.genoox.com/clinical-db/home), and MetaRNN, already integrated within VarSome (48, 49). MetaRNN aggregates 16 functional prediction scores (eg, Sorting Intolerant From Tolerant (SIFT), Polymorphism Phenotyping v2 HumDiv (PolyPhen-2 HumDiv), and Rare Exome Variant Ensemble Learner (REVEL)), 8 conservation metrics (eg, Genomic Evolutionary Rate Profiling (GERP) and phyloP scores based on the 100-way vertebrate alignment (phyloP100way)), and allele frequency from GnomAD version v2.1.1. These platforms were not used as automated classifiers but rather as evidence-retrieval tools, providing access to previous classifications, functional studies (when available), in silico predictions, conservation data, and updated population allele frequencies. Outputs were not applied verbatim. Instead, all evidence was critically reviewed, cross-checked, and manually integrated within the ACMG/AMP framework. Because these resources are continuously updated, the final variant assessment was performed in 2025 using the versions available at that time: VarSome Clinical v13.0.1, InterVar v2.2.1, and Franklin v89 to v92. Likely causal variants were systematically reevaluated and updated in February 2026. For analysis, we retained only P/LP and VUS. All variants identified in POI patients were subsequently screened in the control cohort, and all applied ACMG/AMP criteria are listed in Table S4 (33). Causality was assessed according to each gene's mode of inheritance, and P/LP variants in diagnostic genes were considered likely causal. Additional methodological details are provided in the supplemental material (“Gene variant causality assessment”) (46).

### Statistical analysis

Analyses were performed using SAS software v9.4 (SAS Institute Inc., Cary, NC, USA). Quantitative variables were described using means and standard deviations.

## Results

### Clinical characteristics of patients

A total of 160 women diagnosed with idiopathic POI were included (Fig. 1). All patients had a normal karyotype and no FMR1 premutation. Most were of Caucasian origin (59%), followed by women of North African and Sub-Saharan African descent (32%). Secondary amenorrhea or oligomenorrhea was present in 84%, while PA occurred in 16%. A history of consanguinity was reported in 10%, and familial POI or early menopause in 18%. Among patients with PA, 76% showed absent or incomplete breast development. In women with SA or oligomenorrhea, the mean age at diagnosis was 31.2 ± 7.3 years. Thyroid dysfunction was identified in 26%, including hypothyroidism and/or thyroid autoimmunity (antithyroid peroxidase or antithyroglobulin antibodies: 23.6%) and hyperthyroidism (2.4%). Antiadrenal antibodies were tested in 119 patients and were negative in all cases (Table 1). The mean age of control women at sequencing was 38.7 ± 10.5 years.

**Figure 1:**
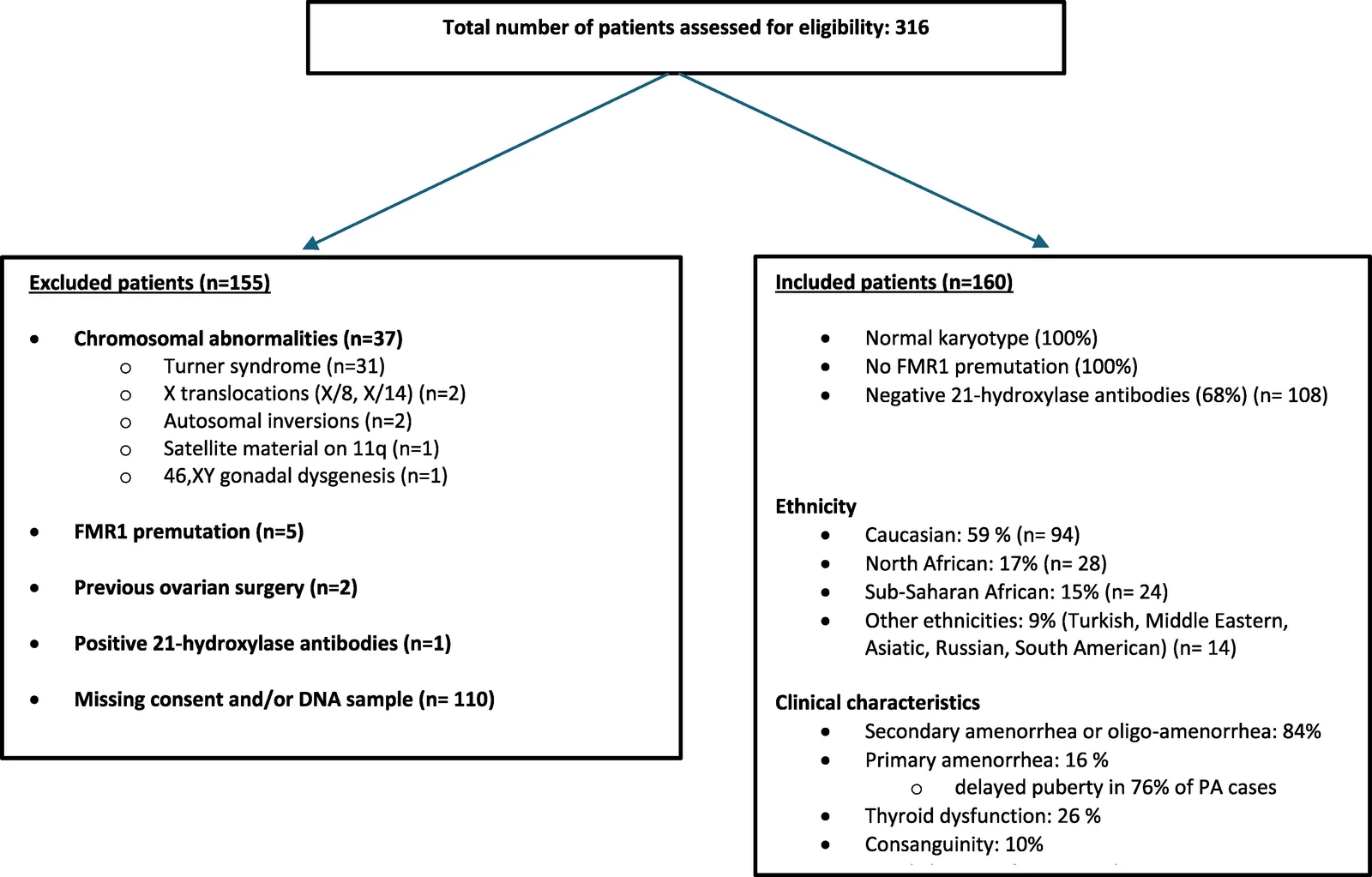
Participant recruitment and study flow diagram. This figure presents the selection process of women with POI and summarizes the final cohort characteristics. Of 316 patients initially screened, 155 were excluded for genetic, clinical, or consent-related reasons. Of the 161 initially included patients, one member of a monozygotic twin pair with POI was subsequently excluded from the analysis. The remaining 160 patients had normal karyotype and no FMR1 premutation, with additional clinical and demographic data shown.

**Table 1:** Clinical and demographic characteristics of patients with premature ovarian insufficiency

Patients	Ethnicity	Amenorrhea (A)	OA/SA (years)	Puberty	Family POI/EM	Consanguinity	Thyroid function	Other patient's particularities
POI-1	North African	Secondary	37	Normal	No	Yes	N	Spontaneous pregnancy 2 years after POI diagnosis
POI-2	North African	Secondary	39	Normal	No	No	N	
POI-3	North African	Primary		Delayed	No	Yes	Hypothroidism	Fibromyalgia, inherited optic neuropathy
POI-4	Turkish	Primary		Delayed	Yes	No	N	Two maternal cousins with the same POI phenotype (PA)
POI-5	Middle Eastern	Secondary	38	Normal	No	No	N, anti-TPO Ab−	
POI-6	Caucasian	Primary		Normal	No	No	Hypothroidism	
POI-7	Sub-Saharan African	Secondary	36	Normal	No	No	N, anti-TPO Ab−	
POI-8	Caucasian	Primary		Delayed	Yes	No	N, anti-TPO Ab−	
POI-9	Sub-Saharan African	Secondary	33	Normal	No	No	N, anti-TPO Ab−	HIV-positive
POI-10	Caucasian	Primary		ND	No	No	N	Incontinentia pigmenti (X linked)
POI-11	Caucasian	Primary		Normal	Yes	No	N, anti-TPO Ab−	
POI-12	Sub-Saharan African	OligoA	37	Normal	No	No	N	
POI-13	Sub-Saharan African	Secondary	22	Normal	Yes	Yes	N	
POI-14	Caucasian	Secondary	30	Normal	Yes	No	Hypothroidism	
POI-15	North African	Secondary	18	Normal	No	Yes	N	
POI-16	Sub-Saharan African	Secondary	33	Normal	Yes	No	N, anti-TG Ab−	
POI-17	North African	Secondary	24	Normal	No	Yes	N	
POI-18	North African	Secondary	39	Normal	Yes	No	N, anti-TPO Ab+	
POI-19	North African	Secondary	28	Normal	No	No	N	
POI-20	Russian	OligoA	30	Normal	No	No	N	Fetus with situs inversus, cousin with heart defect, spontaneous monozygotic twin pregnancy 1 year after POI diagnosis
POI-21	Caucasian	Secondary*^a^*	26	Normal	No	No	N	Sister with breast cancer
POI-22	Caucasian	Secondary	33	Normal	Yes	No	N, anti-TG Ab−	Spontaneous pregnancy 4 months after POI diagnostic
POI-23	North African	Primary		ND	No	Yes	Hypothroidism	
POI-24	North African	Secondary	38	Normal	No	Yes	Hypothroidism	
POI-25	North African	Secondary*^a^*	27	Normal	No	No	N	
POI-26	North African	Secondary	37	Normal	No	No	N, anti-TG Ab−	
POI-27	Caucasian	Secondary	39	Normal	No	No	N, anti-TG Ab−	Breast cancer mother/sister
POI-28	Caucasian	Primary		Delayed	No	Yes	Hypothroidism	
POI-29	Caucasian	Secondary	22	Normal	No	No	N	
POI-30	Caucasian	Primary		Delayed	No	No	N, anti-TPO Ab+	
POI-31	Sub-Saharan African	Secondary	39	Normal	Yes	No	N, anti-TG Ab−	
POI-32	Turkish	Secondary	33	Normal	No	No	N, anti-TPO Ab−,	
							anti-TG Ab−	
POI-33	Caucasian	Primary		Delayed	No	No	N, anti-TPO Ab−	History of uveitis, mother with breast cancer
POI-34	Caucasian	Secondary	32	Normal	No	No	Hypothroidism	AMH 2.8 ng/mL
POI-35	Caucasian	Secondary	31	Normal	Yes	No	Hypothroidism	Cousin with deafness
POI-36	Sub-Saharan African	Secondary	35	Normal	No	No	N, anti-TPO Ab−	
POI-37	Caucasian	Secondary*^a^*	17	Normal	No	No	N, anti-TPO Ab−	Brother with leukemia at age 12
POI-38	Caucasian	Secondary	36	Normal	No	No	Hypothroidism	
POI-39	Caucasian	Secondary	26	Normal	No	No	N, anti-TPO Ab−	
POI-40	Caucasian	Secondary	36	Normal	No	No	N, anti-TG Ab−	
POI-41	Caucasian	Secondary*^a^*	39	Normal	No	No	N	Brother with lymphoma; son and nephew with hand and finger abnormalities
POI-42	Caucasian	OligoA	37	Normal	Yes	No	N	
POI-43	Caucasian	Secondary	30	Normal	No	No	N	Family history of cancer (testicular, breast, leukemia)
POI-44	Caucasian	Secondary	38	Normal	No	No	Hypothroidism	
POI-45	Caucasian	Secondary	21	Normal	No	No	N, anti-TPO Ab−	
POI-46	Caucasian	Primary		Delayed	No	No	N, anti-TPO Ab−	
POI-47	Caucasian	Primary		Delayed	No	No	N, anti-TPO Ab+	
POI-48	Caucasian	Secondary	21	Normal	No	No	N, anti-TPO Ab−	
POI-49	Caucasian	Primary		Normal	Yes	No	N	AMH 1.4 ng/mL, sister with POI and AMH 5.8 ng/mL
POI-50	Caucasian	Secondary*^a^*	16	Delayed	No	No	Hyperthyroidism	
POI-51	North African	Secondary	36	Normal	Yes	No	N	
POI-52	Caucasian	Secondary	27	Normal	No	No	N	Mother with breast cancer
POI-53	North African	Secondary	22	Normal	No	Yes	N, anti-TPO Ab−, anti-TG Ab−	Thyroid cancer at age 32
POI-54	Caucasian	Secondary	34	Normal	No	No	N	
POI-55	Sub-Saharan African	Secondary	30	Normal	No	No	N, anti-TPO Ab−, anti-TG Ab−	
POI-56	Sub-Saharan African	Secondary	36	Normal	Yes	No	N, anti-TPO Ab−	
POI-57	Asian	Primary		Delayed	No	No	N	
POI-58	Caucasian	Secondary	39	Normal	No	No	N, anti-TPO Ab−	
POI-59	North African	Secondary	39	Normal	Yes	No	Hypothroidism	
POI-60	North African	Secondary	37	Normal	No	No	N, anti-TPO Ab−	
POI-61	North African	Secondary	39	Normal	No	No	N, anti-TPO Ab−	
POI-62	Caucasian	Secondary	24	Normal	No	No	N	
POI-63	Caucasian	Secondary	33	Normal	No	No	N, anti-TPO Ab+	Mother with congenital deafness
POI-64	Caucasian	Secondary	28	Normal	Yes	No	Hyperthyroidism	Monozygote twin sister with OA at 28 years; mother and maternal grandmother with EM at 40 years.
POI-65	Caucasian	Secondary	35	Normal	No	No	N	
POI-66	Caucasian	Secondary	33	Normal	No	No	N, anti-TPO Ab−	
POI-67	Caucasian	Primary		Delayed	No	No	Hypothroidism	
POI-68	Caucasian	Secondary	37	Normal	No	No	Hypothroidism	
POI-69	Caucasian	Secondary	32	Normal	No	No	ND	
POI-70	Asian	Secondary	14	Delayed	No	YES	N	Adopted, short stature (1m45)
POI-71	Caucasian	Secondary*^a^*	36	Normal	No	No	Hypothroidism	
POI-72	North African	Secondary	35	Normal	No	No	N, anti-TPO Ab−	
POI-73	North African	Secondary	37	Normal	No	Yes	N, anti-TPO Ab−	Sister with ovarian cancer
POI-74	Caucasian	Primary		Delayed	No	No	N	Breast implants at 20 years due to insufficient breast development despite 5 years of hormone therapy. Paternal half-sister diagnosed with cancer at age 6.
POI-75	Caucasian	OligoA	32	Normal	No	No	N, anti-TPO Ab−	
POI-76	Caucasian	Primary?	15	Normal	No	No	N	One episode of vaginal bleeding at age 15; AMH 7 ng/mL; maternal aunt with late menarche at 18 years
POI-77	North African	Secondary	35	Normal	No	Yes	N	
POI-78	Sub-Saharan African	OligoA	31	Normal	No	No	N, anti-TPO Ab−	
POI-79	Caucasian	Secondary	31	Normal	Yes	No	N, anti-TG Ab+	
POI-80	Caucasian	Secondary	36	Normal	No	No	N, anti-TPO Ab−	
POI-81	Turkish	Secondary	16	Normal	No	Yes	N, anti-TPO Ab−	
POI-82	North African	Secondary	37	Normal	No	No	N, anti-TPO Ab−	
POI-83	Caucasian	OligoA	37	Normal	No	No	N, anti-TPO Ab−	
POI-84	Caucasian	Secondary	37	Normal	No	No	Hypothroidism	
POI-85	North African	Secondary	38	Normal	No	No	N, anti-TPO Ab−	
POI-87	Caucasian	Secondary	38	Normal	No	No	N, anti-TPO Ab−	
POI-86	Caucasian	OligoA	30	Normal	No	No	N	
POI-88	Caucasian	OligoA	33	Normal	No	No	N, anti-TPO Ab−	
POI-89	Caucasian	OligoA	24	Normal	No	No	N, anti-TPO Ab−	AMH 2.4 ng/mL
POI-90	Caucasian	Secondary	20	Normal	No	Yes	N, anti-TPO Ab−	
POI-91	Caucasian	Secondary	20	Normal	Yes	No	N, anti-TPO Ab−	
POI-92	Caucasian	Secondary*^a^*	36	Normal	No	No	N, anti-TPO Ab−	
POI-93	Caucasian	Secondary*^a^*	36	Normal	No	No	N, anti-TPO Ab−	
POI-94	Caucasian	Secondary	39	Normal	No	No	Hyperthyroidism	
POI-95	Caucasian	Secondary	35	Normal	Yes	No	N	
POI-96	Caucasian	Secondary	32	Normal	Yes	No	Hypothyroidism	Psoriasis
POI-97	North African	Secondary	36	Normal	No	No	N, anti-TPO Ab−	
POI-98	Sub-Saharan African	Primary		Delayed	No	No	N	
POI-99	Caucasian	Primary		Delayed	No	No	N, anti-TPO Ab−	Ehlers–Danlos syndrome diagnosed in the patient, her mother, sisters, and maternal family
POI-100	Caucasian	Secondary	27	Normal	Yes	No	N	Unexplained secondary infertility, previous ectopic pregnancy, miscarriage, molar pregnancy, and sister with POI
POI-101	Caucasian	Secondary	17	Normal	No	No	Hypothyroidism	
POI-102	Sub-Saharan African	OligoA	32	Normal	No	No	Hypothyroidism	
POI-103	South American	Primary		ND	No	No	Hypothyroidism	
POI-104	Caucasian	Primary		Delayed	No	No	N	Monozygotic twin sister without POI
POI-105	Sub-Saharan African	OligoA	35	Normal	No	No	N, anti-TPO Ab−	
POI-106	Sub-Saharan African	Secondary	32	Normal	No	No	N, anti-TPO Ab−	
POI-107	Sub-Saharan African	Secondary	30	Normal	No	No	N, anti-TPO Ab−	
POI-108	Sub-Saharan African	OligoA	30	Normal	No	No	N	
POI-109	Sub-Saharan African	Primary		Delayed	No	Yes	Basedow disease	Cousin with deafness
POI-110	Caucasian	Secondary	28	Normal	No	No	N	
POI-111	Caucasian	Primary		Delayed	No	No	N, anti-TPO Ab−	Mother presented 10 miscarriages
POI-112	Sub-Saharan African	Secondary*^a^*	26	Normal	No	No	N, anti-TPO Ab+	Short stature (1m50)
POI-113	Caucasian	Secondary	31	Normal	No	No	N, anti-TPO Ab+	Mother and sister with Hashimoto disease
POI-114	Caucasian	Secondary	29	Normal	No	No	N	
POI-115	Caucasian	Secondary	39	Normal	Yes	No	N, anti-TPO Ab−	
POI-116	Caucasian	Secondary*^a^*	35	Normal	No	No	N, anti-TPO Ab−	
POI-117	North African	Secondary	39	Normal	No	No	N, anti-TPO Ab+	
POI-118	Caucasian	Primary		Delayed	No	No	N	
POI-119	Caucasian	Secondary	38	Normal	No	No	Hypothyroidism	
POI-120	North African	Secondary	35	Normal	No	No	N	
POI-121	Middle Eastern	Secondary	39	Normal	No	Yes	N	
POI-122	North African	Secondary	38	Normal	Yes	No	N	
POI-123	Caucasian	Secondary	27	Normal	No	No	Hypothyroidism	
POI-124	Caucasian	Secondary	19	Normal	No	No	N, anti-TPO Ab−	
POI-125	North African	Secondary	15	Normal	Yes	No	N	
POI-126	Caucasian	OligoA	35	Normal	Yes	No	N	
POI-127	Caucasian	Secondary	36	Normal	No	No	N	
POI-128	Turkish	Secondary	19	Normal	No	No	Hypothyroidism	Mother with Hashimoto disease
POI-129	Caucasian	Secondary	15	Normal	No	No	N	
POI-130	Russian	Secondary	28	Normal	No	No	N, anti-TPO Ab−	
POI-131	Caucasian	Secondary	35	Normal	No	No	Hypothyroidism	Two brothers with deafness, spontaneous pregnancies 7 months after POI diagnosis
POI-132	Sub-Saharan African	Secondary	30	Normal	Yes	No	N, anti-TPO Ab−	
POI-133	Caucasian	Secondary	39	Normal	No	No	N, anti-TPO Ab−	Mother with ovarian cancer
POI-134	Caucasian	Secondary*^a^*	23	Normal	No	No	N, anti-TPO Ab+	
POI-135	Caucasian	Secondary*^a^*	28	Normal	No	No	N, anti-TPO Ab−	
POI-136	Caucasian	Primary		Delayed	No	No	Hypothyroidism	Ovarian biopsy: presence of primordial follicles
POI-137	Asian	OligoA	34	Normal	No	No	N, anti-TPO Ab−	
POI-138	Caucasian	Secondary	31	Normal	No	No	N, anti-TG Ab−	
POI-139	Caucasian	Secondary*^a^*	39	Normal	No	No	N, anti-TPO Ab−	
POI-140	Caucasian	OligoA	34	Normal	No	No	ND	
POI-141	Caucasian	OligoA	36	Normal	Yes	No	N, anti-TPO Ab−	
POI-142	Caucasian	Secondary	30	Normal	No	No	N, anti-TPO Ab−	
POI-143	Caucasian	Secondary*^a^*	34	Normal	Yes	No	N, anti-TPO Ab+	
POI-144	Caucasian	Secondary	32	Normal	No	No	N	
POI-145	Caucasian	Secondary	26	Normal	No	No	N	
POI-146	Asian	Primary		Delayed	No	No	Hypothyroidism	
POI-147	Turkish	OligoA	37	Normal	Yes	No	N, anti-TPO Ab−	
POI-148	Caucasian	Secondary	15	ND	No	No	Hypothyroidism	
POI-149	Caucasian	Secondary	37	Normal	No	No	ND	
POI-150	Caucasian	Secondary	39	Normal	No	No	N, anti-TG Ab−	
POI-E1	Caucasian	Secondary	39	Normal	No	No	N	Mother and maternal grandmother with breast cancer at age 50
POI-E2	Sub-Saharan African	OligoA	36	Normal	No	No	N, anti-TPO Ab−	
POI-E3	North African	Secondary	38	Normal	No	No	N, anti-TPO Ab−	
POI-E4	Sub-Saharan African	Secondary	36	Normal	No	No	N, anti-TPO Ab−	
POI-E5	Caucasian	Secondary	36	Normal	YES	No	N, anti-TPO Ab−	Patient with prolactinoma, mother with POI at age 25
POI-E6	Sub-Saharan African	Secondary	15	Normal	No	No	Hypothyroidism	
POI-E7	Sub-Saharan African	Secondary	33	Normal	No	No	N, anti-TPO Ab−	
POI-E8	Caucasian	OligoA	13	ND	No	No	N, anti-TPO Ab−	Short stature (153 cm)
POI-E9	Sub-Saharan African	Secondary	39	Normal	No	No	N, anti-TPO Ab−	Father with bipolar disorder
POI-E10	North African	Secondary	13	Normal	No	No	N, anti-TPO Ab−	Short stature (147 cm); menarche at 12; slight brachymetacarpia and micrognathia

The table summarizes patient characteristics.

Abbreviations: −, negative; +, positive; anti-TG Ab, antithyroglobulin antibodies; anti-TPO Ab, antithyroid peroxidase antibodies; EM, early menopause; N, normal; ND, no data; OA, oligo-amenorrhea; SA, secondary amenorrhea.

^*a*^Amenorrhea after discontinuation of contraceptive pills.

### Variant landscape

The 155 POI-associated genes were analyzed using Mendeliome sequencing in 150 patients and WES in 10 patients enrolled after June 2022. Overall, 31 patients (19.3%) carried P/LP variants in genes with autosomal dominant (AD), AR, or mixed inheritance patterns. The distribution of variant types and the biological pathways involved are shown in Fig. 2A to 2C. Notably, 37% of P/LP variants affected genes related to DNA repair and meiosis. Figure 2 illustrates the overall variant landscape, including all identified variants, irrespective of their diagnostic relevance.

**Figure 2:**
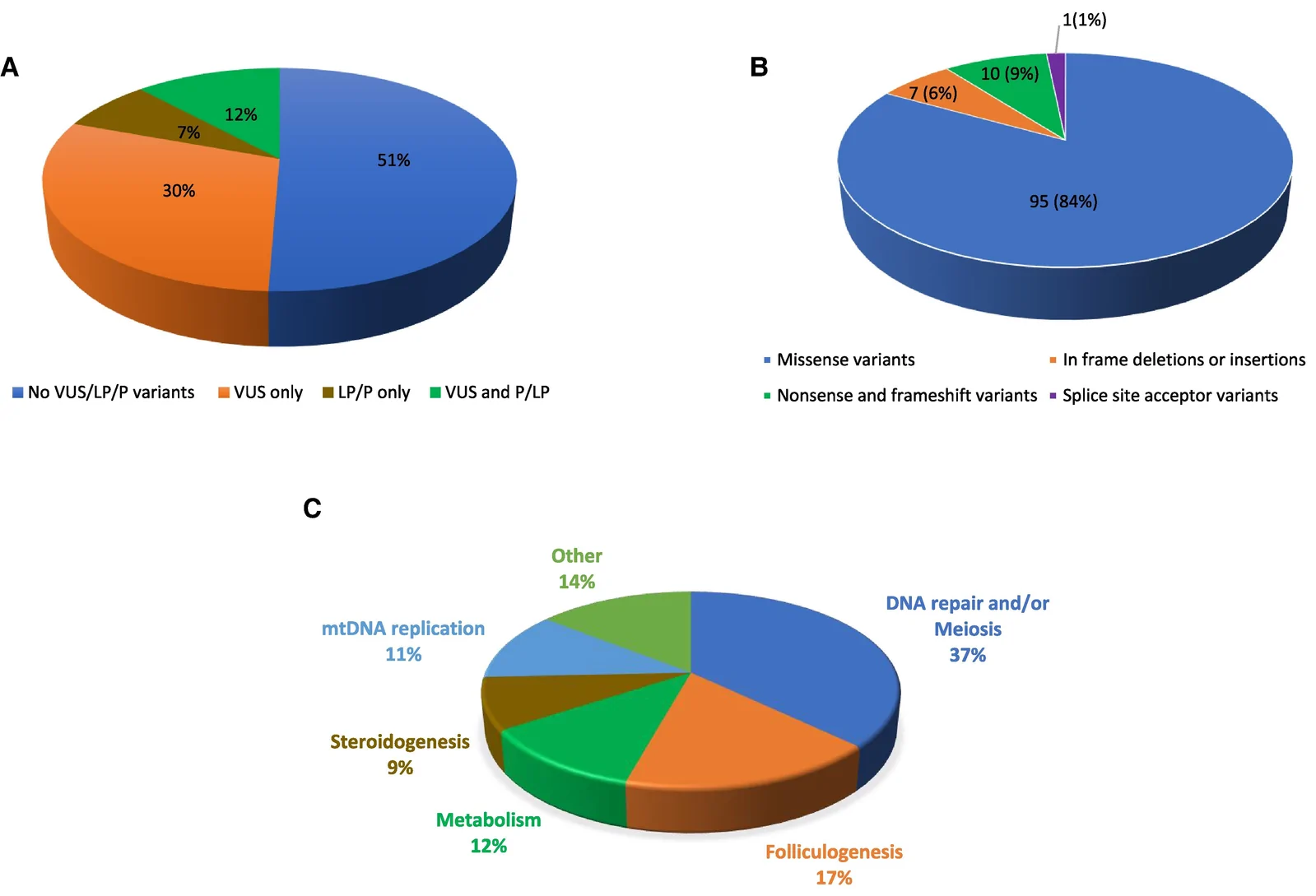
Genetic landscape of variants identified in patients with premature ovarian insufficiency. This figure summarizes the following: (A) Distribution of patients by variant category: among the 160 patients, 81 had no detected variants, 48 carried only VUS, 12 had P/LP variants only, and 19 harbored both P/LP and VUS variants. (B) Distribution of variant types: most variants were missense, which limits direct clinical interpretation and contributes to the high proportion of VUS (not considered diagnostic). (C) Molecular pathways of genes with P/LP variants: these genes are primarily involved in DNA repair and/or meiosis, folliculogenesis, and mitochondrial replication.

Abbreviations: POI, primary ovarian insufficiency; VUS, variant of unknown significance; P, pathogenic; LP, likely pathogenic.

### Diagnostic yield

In total, 113 distinct variants (including VUS and P/LP variants) were identified in 79 patients, corresponding to a variant detection rate of 49.4% (Fig. 2). Thirty-one patients (19.3%) carried P/LP variants, but at the end, only a subset of these (12 patients, 7.5%) were considered clinically relevant. Ten patients were heterozygous carriers of variants in genes with AR inheritance patterns. In 21 patients (13%), we identified P/LP variants consistent with known POI inheritance mechanisms (heterozygous variants in AD or AD/AR genes, or biallelic variants in AR genes). Two LP cis gene variants in DNA polymerase gamma, catalytic subunit (POLG) (POI-8) and homozygote variants in *MRPS22* (POI-13) were not detected in affected relatives and were therefore considered noncausal. Another *POLG* LP variant (POI-137) was also considered likely noncausal, as POLG-related POI has only been reported in the biallelic state for this same variant specifically in the first description of biallelic *POLG* variants causing nonsyndromic POI without neurological disease and the heterozygous mother in that report had a normal menopausal age (50). In the remaining 18 patients (11.3%), 6 carried variants in candidate POI genes that are not considered for causality. The remaining 12 (7.5%) carried likely causal variants in the 50 diagnostic genes. Of these, 7 patients (4.4%) harbored variants in genes with GDR ≥9, namely *FSHR* (POI-49, POI-76), *FANCA* (POI-53, POI-65), *FOXL2* (POI-61), *EIF2B5* (POI-96), and *NR5A1* (POI-135). An additional 5 patients (3%) had variants in genes with GDR score <9 but supported by recent evidence: *CPEB1* (POI-64), *MEIOB* (POI-100), *POLG* (POI-103, POI-149), and *TP63* (POI-74) (21, 22, 36-40). When restricting the analysis to the 40 genes with GDR score ≥9, the diagnostic yield was 4.4%, increasing to 7.5% when the analysis was extended to all 50 retained diagnostic genes (Tables 2 and S4) (33). Further details regarding likely causal variant classification are provided in the supplemental material (“Summary of retained likely causal variants and their ACMG classifications”) (46).

**Table 2:** Summary of gene variants retained as likely causal for POI in 12 patients, with ACMG/AMP classification and allele frequencies

Patient	Gene_symbol	Inheritance	Zygosity	DNA_change	Protein_change	Change_type	ACMG/AMP	ClinVar	GnomAD frequency in female*^a^*	Controls
POI-49	*FSHR*	AR	CHZ	c.1313C>T	p.Thr438Ile	Missense	P	ND	NF	0
POI-49	*FSHR*	AR	CHZ	c.646G>A	p.Gly216Arg	Missense	P	ND	NF^c^	0
POI-53	*FANCA*	AD	HZ	c.2749C>T	p.(Arg917Ter)	Nonsense	P	P	NF^c^	0
POI-61	*FOXL2*	AD/AR	HZ	c.560G>A	p.Gly187Asp	Missense	P	Conflicting	NF	0
POI-64	*CPEB1*	AD/AR	HZ	c.1512C>A	p.(Tyr504Ter)	Nonsense	P	ND	NF^c^	0
POI-65	*FANCA*	AD	HZ	c.3788_3790delTCT	p.(Phe1263del)	In-frame deletion	P	P	0.0000782	0
POI-74	*TP63*	AD	HZ	c.1780C>T	p.(Arg594*^a^*)	Nonsense	P	P	NF^c^	0
POI-76	*FSHR*	AR	CHZ^b^	c.1287T>A	p.(Tyr429Ter)	Nonsense	LP	ND	NF^c^	0
POI-76	*FSHR*	AR	CHZ^b^	c.2031_2032delAG	p.(Arg677SerfsTer31)	Frameshift	LP	ND	NF^c^	0
POI-96	*EIF2B5*	AR/AD	HZ	c.1015C>T	p.(Arg339Trp)	Missense	P	LP/P	0.0000259	0
POI-100	*MEIOB*	AR	CHZ^b^	c.826G>T	p.(Glu276Ter)	Missense	P	ND	0.0000179	0
POI-100	*MEIOB*	AR	CHZ*^b^*	c.905C>T	p.(Thr302Ile)	Missense	VUS (LP)	ND	NF*^c^*	0
POI-103	*POLG*	AD/AR	HZ	c.2246T>C	p.Phe749Ser	Missense	P	P/LP	0.0000433	0
POI-135	*NR5A1*	AD	HZ	c.1310T>A	p.Leu437Gln	Missense	P	P	NF*^c^*	0
POI-149	*POLG*	AD/AR	HZ	c.2209G>C	p.Gly737Arg	Missense	P	Conflicting	0.000675	0

The table summarizes the gene variants identified in individual patients and retained for POI causality in 12 patients, together with their classification according to the American College of Medical Genetics and Genomics/Association for Molecular Pathology (ACMG/AMP) guidelines. More details about ACMG/AMP criteria and results from in silico predictive tools are presented in Table S4 (33). All retained variants considered causal, along with variants of uncertain significance (VUS) identified in diagnostic genes, have been submitted to the Leiden Open Variation Database (LOVD) (Global Variome shared LOVD) (51). The corresponding accession links are provided in Table S4 (33).

Abbreviations: AD, autosomal dominant; AR, autosomal recessive.

^*a*^gnomAD version v2.1.1 (female population); HZ, heterozygous; CHZ, compound heterozygous.

^*b*^Presumed, in-house NGS control cohort of 716 women; LB, likely benign; LP, likely pathogenic; ND, no data.

^*c*^NF: Not found in both male and female gnomAD populations; P, pathogenic; VUS, variant of uncertain significance; VUS+, VUS predicted to be pathogenic by MetaRNN.

### Phenotype stratification

Stratified by phenotype, the diagnostic yield based on the 40 genes with GDR score ≥9 was higher in PA (7.7%, 2/26: POI-49, POI-76) than in SA (3.7%, 5/134: POI-53, POI-61, POI-65, POI-96, POI-135) (Table 2). Incorporating the 10 newly supported genes increased the overall yield to 7.5%, corresponding to 15.4% in PA (4/26: POI-49, POI-74, POI-76, POI-103) and 6% in SA (8/134: POI-53, POI-64, POI-65, POI-96, POI-100, POI-135, POI-149, POI-156) (Table 2).

## Discussion

We report an NGS study in a well-defined cohort of 160 unrelated women with apparently nonsyndromic POI, primarily of Caucasian and North African origin. P/LP variants were identified in 19.3%, consistent with the landscape described in the largest POI cohort to date (22). Using a diagnostic panel based on GDR scoring system (13, 28) and ACMG/AMP criteria (47), the diagnostic yield was 4.4% for the 40 genes with GDR score ≥9 and 7.5% when 10 recently supported genes were added. Yields were higher in PA (15.4%) than in SA (6%), suggesting that monogenic etiologies are more frequent in earlier-onset forms. Although lower than several published NGS yields (14.4-48%) (11, 15, 16, 18, 21, 22, 24, 27, 29-31, 33, 52-55), many prior estimates are inflated due to methodological variability, including acceptance of variants without causality assessment (15, 16), ignoring inheritance constraints (22), counting VUS as causal (31), inclusion of related individuals (21), or reliance on in silico prediction alone (29, 30). These discrepancies highlight the importance of standardized ACMG/AMP interpretation and restricting analysis to clinically validated genes in unrelated POI patients.

In addition to methodological differences, discrepancies between studies also reflect distinct panel design strategies. Our study follows a clinical, evidence-based diagnostic approach, restricting analysis to 155 genes previously reported in association with POI, curated through an extensive literature review and stratified according to gene–disease relationship validity and inheritance patterns.

In contrast, earlier studies adopted a broad exploratory approach using large gene panels ranging from approximately 295 to up to 1000 candidate genes (16, 24, 26). These panels were based on the premise that key genes involved in ovarian biology could potentially contribute to POI. Consequently, such approaches frequently identify multiple variants per patient and support oligogenic models (16, 24). In our study, the variant detection rate was 49.4%, comparable to previously reported panels (48-75%) (16, 21, 24). The detection rate of P/LP variants (19.3%) was also consistent with Ke et al (22) (18.7-23.5%). However, these figures should not be interpreted as a diagnostic yield, as the clinical interpretation requires consideration of both gene relevance to POI and validated modes of inheritance. Although our approach relied on an evidence-based gene prioritization framework (GDR scoring, as adopted by ESHRE FeRGI) to ensure clinical relevance, it should be acknowledged that this framework partly depends on ACMG/AMP-based variant classification and therefore shares some of its inherent limitations in the context of POI. These limitations are further discussed below and underscore the need for continuous refinement and updating of gene–disease relationship validity assessment as new evidence emerges.

### Could specific gene panels improve diagnostic yields in POI?

To date, no predefined gene panel corresponds to a specific POI phenotype; however, in syndromic forms of POI, phenotype-guided in silico panels derived from WES or other NGS data offer a flexible strategy that tailors gene selection to each clinical presentation, orienting analysis toward neurological, neurometabolic, mitochondrial, skeletal, immunometabolic, or disorders of sex development (DSDs) associated genes (8, 9, 12, 13, 26), which accounts for the markedly higher diagnostic yields observed in syndromic compared with nonsyndromic POI (21). The same applies to meiotic genes that cause POI and are shared with nonobstructive azoospermia highly informative in suspected meiotic failure (56-60) whereas separating panels according to pubertal status or menstrual presentation (PA, SA, oligomenorrhea) is likely less clinically meaningful since identical pathogenic variants may span the entire reproductive continuum (20, 61).

### Genotype–phenotype correlation in POI

Genotype–phenotype correlation in POI is highly complex and cannot be predicted from gene function alone. Clinical variability reflects variant type and location, allelic dosage, and probable oligogenic or epigenetic modifiers, producing wide differences in penetrance and expression even for the same variant across unrelated individuals. *CPEB1* illustrates this complexity particularly well. *CPEB1* is essential for meiotic progression and folliculogenesis and comprises a N-terminal regulatory region, 2 RNA-recognition motifs (RRMs), and a C-terminal zinc-binding domain (62, 63). Variants affecting the N-terminal region, the RRMs, or producing large gene deletions typically result in severe POI (18, 64, 65), whereas C-terminal truncations despite impairing part of the RNA-binding function are more often associated with SA rather than PA (22). In our cohort, the c.1512C>A (p.Tyr504Ter) variant truncates the C-terminal RNA-binding domain. It segregated in a family with a remarkably coherent reproductive pattern: the proband developed SA at age 28; her monozygotic twin carrying the same variant presented with oligo-amenorrhea at the same age; her mother and maternal grandmother, although not genetically tested, had early menopause at 40 years. However, the origin of the variant remains uncertain, as parental genotyping could not be performed. The variant may have been inherited from the father or arisen de novo in the monozygotic twins. The family history may also suggest an underlying genetic susceptibility, potentially consistent with a digenic or oligogenic context. Alternatively, the variant could have been inherited from the maternal lineage and expressed with variable expressivity, leading to a spectrum of phenotypes ranging from early menopause or late-onset POI to POI manifesting at 28 years. *EIF2B5* biallelic pathogenic variants cause vanishing white matter (VWM) disease with associated POI (13, 64). However, different heterozygous *EIF2B5* variants such as p.Cys112Trp (64) and p.Val85Glu (22) have been reported in at least 5 women with isolated, nonsyndromic POI without neurological involvement. Our heterozygous p.Arg339Trp case affects a conserved residue previously reported in VWM disease (UniProtKB Q13144, variant ID: VAR_012330), supporting its functional relevance and a model in which biallelic variants cause syndromic POI, whereas heterozygous variants may contribute to isolated POI. *FANCA* plays a dual role in interstrand cross-link repair and meiotic double-strand break repair (66, 67). Although biallelic *FANCA* variants cause Fanconi anemia with POI in approximately 65% of affected women (67, 68), heterozygous *FANCA* variants have been linked to isolated POI in different reports and in mouse models (66-69). Our 2 heterozygous cases (c.3788_3790delTCT and c.2749C>T) add to this growing evidence for a dosage-sensitive ovarian phenotype. *FOXL2* variants produce a broad phenotypic spectrum from isolated POI to blepharophimosis-ptosis-epicanthus inversus syndrome (BPES types I and II) (70-75). The ovarian-specific effect of c.560G>A (p.Gly187Asp) identified in Tunisian patient diagnosed with POI at 27 was functionally demonstrated by altered *FOXL2* autoregulation with preserved eyelid-specific OSR2 activation (76). Our patient with the same variant developed SA at 39 years after 3 spontaneous pregnancies, illustrating the profound age-of-onset variability even for a same gene variant (77-80). *FSHR* biallelic inactivating variants classically cause hypergonadotropic hypogonadism with preserved ovarian reserve the so-called “FSH-resistant ovary” phenotype (19, 81-84). We have previously reported segregation data and in vitro functional assays for the variants identified in patient POI-49 (19). In patient POI-76, the presumed trans truncating variants were associated with preserved anti-Müllerian hormone (AMH) levels but variable menstrual patterns, findings consistent with partial vs complete loss of FSHR function. The estrogen-withdrawal bleeding observed in this case may mimic SA (19). *MEIOB* is essential for meiotic recombination (59, 85-87). Although human data remain limited, the few available reports consistently associate biallelic *MEIOB* variants with a distinctive reproductive profile characterized by infertility, recurrent pregnancy loss including molar pregnancy and subsequent progression to POI (36, 87). Our presumed compound heterozygous case (p.Glu276Ter + p.Thr302Ile) aligns closely with this emerging *MEIOB*-specific phenotype. *NR5A1* is a key AD gene for 46,XY disorders of sex development (DSD) and 46,XX POI, with more than 218 reported variants but no reliable genotype–phenotype correlation despite extensive data (13, 88-90). Its marked intrafamilial and interfamilial variability from severe DSD to isolated POI or asymptomatic carriage reflects incomplete penetrance, variable expressivity, and probable digenic/oligogenic interactions (89, 91-94). In our cohort, the previously reported pathogenic variant c.1310T>A (p.Leu437Gln) (90), located in the ligand-binding domain essential for transcriptional activity through phospholipid and cofactor interactions (95, 96), was identified in a woman with nonsyndromic POI who developed SA at 28 and was referred for counseling regarding familial POI risk and possible 46,XY DSD in offspring. *POLG*-related POI is classically syndromic, with SA often preceding progressive external ophthalmoplegia and parkinsonism (97). Dominant variants in the catalytic polymerase domain such as p.Arg953Cys, p.Tyr955Cys, and p.Arg943His impair mitochondrial DNA (mtDNA) replication (97-99). By contrast, nonsyndromic POI has been associated with variants across multiple *POLG* regions, including the exonuclease domain, the polymerase domain, and the accessory-interaction determinant/interdomain (AID/IP) spacer, a structurally critical region mediating *POLG–POLG2* interactions (100-102). In our cohort, p.Gly737Arg (SA at 37) and p.Phe749Ser (PA) both mapped to this AID/IP spacer, near the well-established pathogenic hotspot p.Trp748Ser (101). Overall, these findings highlight the extensive allelic and phenotypic heterogeneity of *POLG*-related ovarian dysfunction, encompassing both syndromic and nonsyndromic presentations. Finally, *TP63* variants disrupt oocyte quality control and may cause isolated PA/SA or syndromic ectodermal disease (22, 37-40, 103, 104). Our adolescent PA case without limb-mammary features reinforces this reproductive-restricted presentation.

### Main explanations for the limited diagnostic yield in POI

Despite the high prior probability of a genetic etiology in our cohort, the diagnostic yield remained relatively low whether restricting analysis to established POI genes or extending it to broader candidate lists. However, it is important to highlight that interpreting nontruncating variants in POI requires particular caution, especially in later adult-onset cases, which represent the most prevalent POI phenotype. (1) Many causal genes remain unidentified: Family-based studies consistently show much higher yields (17, 21, 64), but these approaches are often impractical in POI because of late onset and frequent unavailability of parental DNA. (2) POI often reflects oligogenic, polygenic, or epigenetic influences: Such multilayered genetic architecture can markedly reduce penetrance and increase phenotypic variability, preventing clear diagnostic assignments. (3) Reduced performance of ACMG/AMP criteria in POI: ACMG/AMP criteria were developed for highly penetrant Mendelian disorders and therefore underperform in POI, a condition with incomplete penetrance, late onset, and high prevalence (3.5%): (a) Limitations of ACMG population-based criteria (BS1, BS2, PM2, PS4) (47). Similar to gnomAD, our Highlander internal controls include individuals without severe congenital disease but lack reproductive information, capturing only sex and age (105). Thus, both datasets may include women with undiagnosed, late-onset, or presymptomatic POI, artificially inflating the frequency of truly pathogenic alleles. However, our control cohort may reflect more accurately the multiethnic Brussels population. (b) Evidence of low penetrance. Shekari et al (25) reported that 99.9% of heterozygous protein-truncating variants across 105 POI genes including 40 AD genes were present in reproductively normal UK Biobank women. However, because POI in that cohort relied on self-reported menopause before age 40, the phenotype likely reflects later-onset or SA-type POI. Furthermore, biallelic genotypes were not evaluated, preventing conclusions about recessive and maybe early-onset severe POI. In addition, the study did not exclude late truncating (stop-gain) variants unlikely to impair protein function or splice-site variants without demonstrated functional impact. (c) Ovarian reserve as a major modifier of penetrance: Large interindividual variability in baseline ovarian reserve (AMH values ranging from extremely low to >36 ng/mL) could modify phenotypic expression, such that the same genetic variant can manifest as PA, early SA, POI in the early thirties, early menopause, or even a normal menopausal age. In addition, natural variation in age at menopause in the general population, extending to the lower extreme of the distribution, may further contribute to this variability. This could explain low penetrance reported by Shekari et al (25) and limits ACMG segregation-based criteria (PP1 and PP4) (47). (d) High allele frequency does not necessarily exclude pathogenicity for late POI onset: Adult-onset POI preserves fertility before disease onset, thereby reducing negative selection (20), unlike early-onset POI and severe life-threatening Mendelian disorders in which pathogenic variants are efficiently purged from the population. Moreover, control datasets may include women with undiagnosed POI or individuals with initially high ovarian reserve who never manifest clinical insufficiency, artificially inflating allele frequencies. Consequently, population-based frequency thresholds cannot reliably exclude pathogenicity particularly for functionally supported, nontruncating variants such as several *NOBOX* alleles highlighting the need for cautious interpretation in the context of variably penetrant, adult-onset POI (20). (e) ACMG misclassification examples: *NOBOX* variants with strong functional evidence were down-classified because of frequency thresholds (20), while *NR5A1* shows poor concordance between ACMG class and clinical severity, with pathogenic, VUS, and likely benign (LB) variants distributed across the full DSD spectrum (88). (f) Underestimated heterozygous effects in recessive genes: Even recessive genes like *STAG3* responsible for severe early-onset POI with PA and delayed puberty can produce later-onset POI or early menopause in heterozygous carriers (106). The absence of POI phenotypes in heterozygous carriers in AR genes may reflect underrecognition of later onset phenotype rather than true absence of effect. For these reasons—and to ensure transparency—we report all heterozygous P/LP and VUS variants while clearly distinguishing them from diagnostic findings. (4) Bias introduced by filtering out predicted low-impact variants: Filtering out synonymous or nondisruptive variants may remove biologically relevant alleles. Synonymous variants can alter mRNA structure, splicing, translation, or miRNA binding (107), and similar regulatory effects are well documented in oncology (108). Before ACMG implementation, such variants were reported more frequently, helping explain discrepancies in historical reports for *NOBOX* and *NR5A1* (20, 88). Although truly causal low-impact variants likely represent a minority as illustrated by *NR5A1* likely benign/benign (LB/B) variants enriched in severe 46,XY DSD (∼90%) (88), careful reevaluation of remaining VUS/LB/B variants is warranted, especially when functional evidence is lacking.

## Conclusion

Targeted NGS of 155 POI-associated genes identified P/LP variants in 19.3% of patients. When restricting analysis to the 50 most relevant POI genes and applying ACMG/AMP criteria aligned with appropriate inheritance models, the diagnostic yield ranged from 7.5% in the overall cohort to 15.4% in women with PA. Given the homogeneity of our cohort and the use of a curated diagnostic gene list, these values likely represent a more accurate estimate of the true contribution and inherent limitations of monogenic testing in unrelated, nonsyndromic POI.

## Data Availability

All data supporting the findings of this study in POI patients are provided within this article. Relevant genetic variants identified in this study have been submitted to the Leiden Open Variation Database (LOVD) (51) and are publicly available. The corresponding LOVD links are provided in Table S4 (33). Due to ethical and privacy restrictions associated with human genomic data, deidentified raw next-generation sequencing (NGS) data from POI patients and control women are not publicly available. These data may be made available by the corresponding author upon justified request, following appropriate data access procedures and in accordance with participant consent and applicable regulations.

## Current affiliation

Marc Abramowicz: Genetic Medicine Division, Diagnostics Department, Geneva University Hospitals (HUG), Rue Gabrielle Perret Gentil 4, Geneva 1205, Switzerland. Julie Désir: Institut de Pathologie et de Génétique (IPG), Gosselies 6041, Belgium.
